# Visual tools for 3D plasma simulation: GPU-based isosurface extraction and particle-based rendering

**DOI:** 10.1371/journal.pone.0359294

**Published:** 2026-10-16

**Authors:** Jong-Hyun Kim, Mintae Na, Junho Kim, Sejin Yun, Jung Lee, Sun-Jeong Kim

**Affiliations:** 1 College of AI and Convergence (Dept. of Artificial Intelligence, Design Technology), Graduate School of Electrical and Computer Engineering, Inha University, Michuhol-gu, Incheon, South Korea; 2 Department of Convergence Software, Hallym University, Chuncheon, South Korea; 3 Department of Computer Engineering, Hanbat National University, Yuseong-gu, Daejeon, South Korea; Donghua University, CHINA

## Abstract

This paper presents a GPU-accelerated visualization framework for interactive exploration of fusion-plasma simulation data represented as scalar fields and particle trajectories. The primary technical contribution is a capacity-safe isosurface-extraction pipeline that combines inclusive slab partitioning with a two-pass counting and exact allocation strategy. This design prevents slab-boundary omissions and output-buffer overflow while supporting large scalar grids. The generated positions and normals are written directly to CUDA–OpenGL interoperable buffers, enabling the standalone renderer to display explicit isosurfaces without an intermediate CPU-side copy. The extracted geometry and scalar-volume data are additionally integrated into Unreal Engine through a separate staging interface. This integration provides a unified environment for volume rendering, explicit-isosurface inspection, particle-trajectory visualization, clipping, and VR-based spatial exploration. These engine-side functions are used as interaction and visualization mechanisms rather than being presented as new rendering algorithms. Experiments on electromagnetic-potential fields with resolutions up to 512^3^ demonstrate stable isosurface extraction across different isovalues. In the most geometrically complex tested case, the method generates approximately 42.81 million vertices in 44.7 ms, uses less than 11 GB of GPU memory, and renders the resulting geometry at 28.6 FPS. The results indicate that the proposed framework provides a practical explicit-surface visualization path for large plasma datasets while complementing direct volume rendering and established scientific-visualization toolkits.

## Introduction

Fusion plasma simulations involve extremely large-scale interactions among electromagnetic fields, fluids, and particles, and both grid and temporal resolutions continue to grow rapidly. As a result, the data volume frequently reaches hundreds of gigabytes to several terabytes, comprising mixtures of isosurfaces, scalar fields (e.g., potential, pressure, charge density), and trajectory/streamline-based particle datasets. Visualizing such complex and massive data in 3D is essential for understanding morphological phenomena–-such as boundary structures, instabilities, and transport paths–-yet the workflow in many research environments remains fragmented into *offline preprocessing* → *mesh extraction* → *file export* → *offline viewer loading*, which inherently limits interactive analysis.

In particular, examples of real-time (interactive-rate) 3D visualization for plasma data are rare. This is primarily due to the following factors: (1) for high-resolution grids (e.g., 512^3^ or above), isosurface extraction methods such as Marching Cubes or Flying Edges incur substantial *memory and bandwidth bottlenecks*; (2) the extracted meshes often contain tens of millions of vertices, creating additional bottlenecks at the *rendering stage*; and (3) when particle-based visualization (trajectory/streamline tracing) is incorporated, host-to-device (H2D) and device-to-host (D2H) data transfers between the computation and visualization stages can introduce substantial overhead. Consequently, a common pattern has been “simulation on the GPU, visualization via CPU/files/external viewers,” forcing users to rely on batch-style, offline results instead of interactive exploration.

To address this gap, we propose a GPU-centered plasma visualization pipeline and game-engine integration framework that minimizes CPU-side transfers in the standalone rendering path while supporting engine-based visualization through a separate staging interface. Specifically, we integrate isosurface extraction–implemented via a capacity-safe Marching Cubes algorithm–with particle-based rendering (particle/trajectory tracing) into a single lightweight GPU workflow. The key idea is to employ slab tiling and inclusive slab indexing to eliminate boundary cracks, and to adopt a two-pass pipeline (classification/scan → exact allocation) that guarantees crack-free isosurfaces and prevents buffer overruns even at high grid resolutions. The generated mesh and normals are streamed zero-copy into VBOs through CUDA–OpenGL interop, allowing them to be rendered immediately without any CPU round-trip.

Furthermore, the proposed pipeline is integrated with the commercial game engine Unreal Engine, enabling an end-to-end workflow that unifies volume → isosurface mesh → particle tracing within a single editing and execution environment. Engine-side compute-based particle tracing (streamlines/pathlines) combined with color, size, and lifetime mappings allows rapid identification and emphasis of plasma trajectories. Parameter sweeps (ISO threshold, seed/step), LOD control, and snapshot/export (OBJ) functions streamline the entire analysis workflow, from data loading to reporting.

In summary, the main contributions of this paper are as follows:

- A slab-based, two-pass GPU isosurface-extraction pipeline that combines exact output allocation with boundary-consistent indexing for large scalar grids.
- A tightly coupled extraction and rendering workflow that writes the generated geometry directly to an interoperable graphics buffer.
- An Unreal Engine visualization environment that integrates scalar-volume rendering, explicit isosurfaces, particle trajectories, clipping, parameter control, and VR-based spatial inspection.

## Related work

This section reviews representative research across key axes of large-scale 3D scientific visualization–-isosurface extraction, GPU acceleration and memory/bandwidth optimization, particle (trajectory/streamline) visualization, screen-space enhancement techniques, and in-situ/engine integration.

### Explicit isosurface extraction

The classical Marching Cubes (MC) algorithm by Lorensen and Cline introduced a simple procedure in which the signs of the eight cell corners are classified to generate triangles using a case table [1]. However, the standard MC formulation can produce topological discontinuities and holes due to ambiguities in cell-internal interpolation. The Asymptotic Decider addresses such cases by resolving ambiguous configurations using the asymmetry of the internal contour [2]. Chernyaev’s MC33 systematically enumerates 33 disambiguated cases, ensuring improved topological consistency [3], while Dual Contouring preserves sharp features by utilizing edge Hermite data (intersection points and normals) [4]. Recent approaches such as SurfaceNets and Flying Edges emphasize boundary locality and parallelism. Unlike topology-oriented extensions such as MC33 and Dual Contouring, the present work focuses on boundary-consistent slab processing and exact GPU output allocation for engine-integrated visualization.

### Direct volume rendering and implicit isosurfaces

Direct volume rendering maps scalar values to optical properties through transfer functions and accumulates color and opacity along viewing rays without explicitly constructing surface geometry. GPU-based ray casting has become a standard approach for interactive volume visualization because it supports flexible transfer-function control, clipping, gradient-based shading, and inspection of internal structures. Implicit isosurfaces can also be detected during ray traversal by locating scalar-field crossings at selected isovalues. This approach avoids explicit triangle storage and allows multiple isosurfaces to be rendered within the same ray-casting framework.

In the present system, direct volume rendering and explicit isosurface extraction serve different purposes. Volume ray casting is used in the Unreal Engine environment for interactive inspection of the complete scalar field, including transfer-function-based emphasis and clipping-plane exploration. In contrast, the Marching Cubes pipeline produces explicit geometry that can be rendered through conventional rasterization, combined with reactor meshes, manipulated as a geometric object, and exported for subsequent use. The two approaches are therefore treated as complementary rather than competing visualization modes.

### GPU-based large-scale isosurface extraction

For large grids, the main computational challenges arise from memory-bandwidth requirements, branching during cell classification and table lookup, and management of output geometry whose size is not known in advance.

HistoPyramids-based Marching Cubes determines output locations through hierarchical counting and stream compaction, thereby avoiding uncontrolled dynamic output. Prefix-sum-based GPU implementations similarly compute per-cell primitive counts before geometry generation. Flying Edges reorganizes the extraction process around grid edges to improve memory locality and reduce redundant computation. VTK-m provides data-parallel abstractions and manages intermediate and output storage through its execution framework, supporting portable isocontouring across CPUs and GPUs.

These approaches demonstrate that safe output-buffer management is already well established in GPU-based isosurface extraction. The present work therefore does not claim that existing methods fail to handle memory safely. Instead, our contribution is a lightweight CUDA implementation in which slab-local counts are accumulated into an exact global allocation, inclusive slab boundaries maintain consistent cell coverage, and the generated vertices and normals are written directly to an interoperable rendering buffer. This design specializes existing counting and compaction principles for tightly coupled extraction, rendering, and engine-oriented visualization within a compact single-GPU workflow.

### GPU interoperability for visualization

Graphics–compute interoperability enables GPU-generated data to be consumed directly by a rendering API without an intermediate CPU copy. CUDA–OpenGL interoperability has been widely used for GPU-based geometry generation, particle systems, and scientific visualization. In this work, interoperability is used as an implementation mechanism for transferring the output of isosurface extraction directly to the OpenGL rendering stage. Detailed buffer-registration and synchronization procedures are described in the Proposed Framework section.

### Shading and screen-space techniques

In scientific visualization, conventional Lambertian shading may provide insufficient contrast when surface orientation varies substantially across the view. Wrapped diffuse shading extends the illuminated region by shifting and rescaling the Lambertian term, while camera-aligned lighting places the light direction near the viewing direction to provide relatively consistent illumination over view-facing structures. Hemisphere lighting and screen-space ambient occlusion can further improve the perception of surface orientation, concavity, and local depth. In this work, we use camera-aligned two-sided diffuse shading as a conventional visualization configuration, together with optional SSAO for enhanced local contrast. These components are used only to improve surface readability and are not presented as novel shading techniques.

### Particle (advection) and streamline visualization

In plasma and fluid analysis, particle trajectories and streamlines play a complementary role to scalar isosurfaces. Laramee et al. established the classical taxonomy of texture-based, geometry-based, and feature-based flow visualization techniques [5,6], and McLoughlin et al. surveyed two decades of integration-based and geometric flow visualization methods [7]. More recently, particle advection using VTK-m has been employed in large-scale simulations due to its performance portability [8]. Our pipeline is designed so that particle visualization can be seamlessly combined within the same frame (with GUI-controlled seeds and step size, and color/size/lifetime mappings), and when integrated with Unreal, it is executed through the engine’s compute pathway.

### In-situ and commercial engine (unreal engine) integration

In-situ visualization performs analysis and rendering concurrently with the simulation (in memory), reducing disk I/O bottlenecks and enabling interactive feedback. ParaView Catalyst provides a lightweight API/adapter for in-situ coupling [9], and VisIt’s Libsim offers a similar interface for simulation-driven visualization [10–13]. SENSEI proposes a generic in-situ interface enabling compatibility with various backends such as Catalyst and Libsim [14,15], while Ascent demonstrates an interactive in-situ pipeline through a lightweight visualization library [16]. This paper explores how such in-situ workflows can be coupled with a commercial engine (Unreal), which offers high-quality rendering, extensive editing workflows, and an established VR ecosystem. However, bridging scientific data with the engine requires addressing technical challenges such as *large-volume streaming, precise vertex/normal management, and analysis-oriented shading*. By unifying volume extraction, isosurface generation, and particle tracing inside the engine, our framework accelerates the exploration of plasma trajectories.

## Proposed framework

This section describes the proposed pipeline in the following order: problem setup and notation, capacity-safe slab-based Marching Cubes, zero-copy rendering, Unreal Engine integration, and implementation parameters and limitations.

**Rationale for Explicit Isosurface Extraction.** Implicit isosurface raycasting is generally more memory-efficient when the objective is limited to direct visualization of one or more isovalues from a resident scalar volume. It avoids explicit triangle generation and can compute smooth normals directly from volume gradients. We therefore do not claim that Marching Cubes is universally more efficient than implicit raycasting.

In this work, however, the extracted surface is used as an explicit geometric asset rather than only as an intermediate rendering representation. The generated geometry is streamed to an OpenGL vertex buffer, imported into the Unreal Engine rendering pipeline, combined spatially with reactor geometry, manipulated using standard mesh-based interaction tools, and optionally exported as an OBJ file. These operations require accessible vertex and normal data and are difficult to support through a purely implicit raycasting representation without an additional surface-extraction stage.

Accordingly, the purpose of the proposed capacity-safe Marching Cubes pipeline is not to replace implicit volume raycasting, but to provide a robust and interactive explicit surface-extraction path for workflows that require reusable geometric output. For visualization tasks that require only direct inspection of scalar volumes or simultaneous display of multiple isovalues, implicit raycasting remains a more appropriate alternative.

### Problem setup and notation

Given an input volume $V:\{0,...,N_{x}\;-\;1\}\;\times \;\{0,...,N_{y}\;-\;1\}\;\times \;\{0,...,N_{z}\;-\;1\}\to \mathbb{R}$ and an isovalue τ∈ℝ, Marching Cubes (MC) computes a case index c∈{0,...,255} by evaluating the sign configuration of the eight corner samples of each cell (*i*,*j*,*k*), and then generates up to five triangles (up to 15 vertices) according to the case table. The linear index is defined as


$$\begin{array}{c} lin(x,y,z)=(z\;N_{y}+y)N_{x}+x, \end{array}$$ (1)

where numVerts[*c*] denotes the number of vertices associated with case *c*, and triTable[c][·] stores the edge–triangle listings. The symbols used in this section are summarized in Table 1.

**Table 1:** Symbols used in this Section ‘Proposed Framework’.

Symbol	Definition
$N_{x},N_{y},N_{z}$	Grid dimensions (samples)
(*i*,*j*,*k*)	Cell index, $0\;\leq \;i\;\leq \;N_{x}\;-\;2$ etc.
τ	Isovalue
*c*	MC case index (0..255)
numVerts[*c*]	# of triangle vertices for case *c*
triTable[c][·]	Triangle edge list for case *c*
${𝒦}_{s}$	Valid cells of slab *s* (inclusive)
$N_{s}$	# of vertices on slab *s* (Pass-1)
*N*	Global # of vertices; $N=\sum_{s}N_{s}$
offset[ℓ]	Exclusive prefix-sum per cell ℓ
base(*s*)	Slab base offset, $\sum_{t<s}N_{t}$

### Capacity-safe tiled marching cubes

*Inclusive slab indexing.* We partition the *z*-axis into slabs of thickness *S*, and define the set of valid cells for slab *s* as the *closed interval* ${𝒦}_{s}=\{\;k|k_{begin}^{\left(s\right)}\leq k\leq k_{end}^{\left(s\right)}\;\}$. This *inclusive* definition ensures that the *k* + 1 corner access required by MC remains valid even at slab boundaries, thereby eliminating *boundary omissions/duplications* and cracks caused by off-by-one inconsistencies.

*Two-pass (classify/scan* → *generate).* The key idea is to first determine *exactly how many vertices will be generated*, and then allocate the interop VBO *exactly* to that size. In Pass 1, for each slab *s* and each cell $\left(i,j,k\in {𝒦}_{s}\right)$, we compute the case index *c* and $n_{ijk}=numVerts[c]$, and compute an *exclusive* prefix-sum offset[ℓ] over the slab’s linear index ℓ. This yields the slab-level total $N_{s}=\sum n_{ijk}$ and the global total $N=\sum_{s}N_{s}$. The VBO is then allocated with *exactly N* vertex capacity. In Pass 2, triangles are written *directly* into the VBO at the address $base(s)=\sum_{t<s}N_{t}+offset[\ell ]$. Winding is enforced to be counterclockwise, and the face normal


$$\begin{array}{c} 𝐧=\frac{\left({𝐩}_{1}-{𝐩}_{0}times({𝐩}_{2}-{𝐩}_{0}\right)}{\left\|({𝐩}_{1}-{𝐩}_{0})\times ({𝐩}_{2}-{𝐩}_{0})\right\|} \end{array}$$ (2)

is duplicated at the three triangle vertices for flat-shaded rendering. For two-sided rendering, the fragment shader reverses the surface normal for back-facing fragments before evaluating the diffuse term.

*Algorithm overview.* The complete extraction procedure is summarized in Algorithm 1.

**Algorithm 1 Capacity-safe tiled Marching Cubes algorithm using inclusive slab indexing and a two-pass (classify/scan → generate) strategy. The method computes exact vertex counts per slab to allocate GPU buffers safely and generates crack-free isosurfaces by eliminating boundary inconsistencies and buffer overruns.**

**Require:** Volume *V*, isovalue τ, inclusive slab ranges $\left\{[k_{b}^{\left(s\right)},k_{e}^{\left(s\right)}]\right\}$

1: N←0 ▷ global vertex count

2: **for** each slab *s* **do**

3: **for** each cell (*i*,*j*,*k*) with $k\in [k_{b}^{\left(s\right)},k_{e}^{\left(s\right)}]$ **do**

4: c←caseIndex(V,i,j,k,τ)

5: $n_{ijk}\leftarrow numVerts[c]$

6: ${counts}_{s}[lin(i,j,k)]\leftarrow n_{ijk}$

7: **end for**

8: ${offset}_{s}\leftarrow exclusive\_scan({counts}_{s})$

9: $N_{s}\leftarrow {offset}_{s}[last]+{counts}_{s}[last]$

10: base[s]←N ▷ base write offset for slab *s*

11: $N\leftarrow N+N_{s}$

12: **end for**

13: **Allocate** interop VBO (pos/nrm) with capacity *N*

14: **for** each slab *s* **do**

15: **for** each cell (*i*,*j*,*k*) with $k\in [k_{b}^{\left(s\right)},k_{e}^{\left(s\right)}]$ **do**

16: c←caseIndex(V,i,j,k,τ) ▷ or reuse if cached

17: **if** numVerts[c]≠0 **then**

18: edgeVerts[0..11]←edge_lerp(V,i,j,k,τ)

19: $dst\leftarrow base[s]+{offset}_{s}[lin(i,j,k)]$

20: **for** t←0 **to** numVerts[c]−1 **step** 3 **do**

21: $\left(e_{0},e_{1},e_{2})\leftarrow triTable[c][t..t+2\right]$

22: **if** *e*_0_ < 0 **or** *e*_1_ < 0 **or** *e*_2_ < 0 **then**

23: **break**

24: **end if**

25: ${𝐩}_{0},{𝐩}_{1},{𝐩}_{2}\leftarrow edgeVerts[e_{0}],edgeVerts[e_{1}],edgeVerts[e_{2}]$

26: $𝐧\leftarrow normalize(({𝐩}_{1}-{𝐩}_{0}times({𝐩}_{2}-{𝐩}_{0}))$ ▷ CCW

27: $pos[dst:dst+2]\leftarrow \{{𝐩}_{0},{𝐩}_{1},{𝐩}_{2}\}$

28: nrm[dst:dst+2]←{𝐧,𝐧,𝐧}

29: dst←dst+3

30: **end for**

31: **end if**

32: **end for**

33: **end for**

**Correctness and computational cost.** The inclusive slab ranges ensure that every valid cell is processed exactly once, including cells adjacent to slab boundaries. Because shared edges use the same scalar samples and isovalue, their interpolated intersection positions remain consistent across neighboring slabs. For $M=(N_{x}-1)(N_{y}-1)(N_{z}-1)$ cells, both passes require *O*(*M*) time. The additional storage consists of the slab-local count and offset arrays, while the output buffers are allocated according to the exact number of generated vertices.

### Zero-copy streaming and visualization-oriented shading

**Interop Rendering.** After CUDA finishes writing the generated positions and normals, the interoperable buffers are unmapped and consumed directly by the OpenGL rendering pipeline. Standard vertex-attribute binding and triangle rasterization are used to render the extracted geometry. Two-sided normal handling, camera-aligned diffuse shading, and optional SSAO are applied as established visualization components. Because these operations follow standard CUDA–OpenGL and GLSL practice, low-level API calls are omitted for brevity.

The current implementation assigns one face normal to each generated triangle, resulting in flat-shaded visualization rather than gradient-based smooth shading. In contrast, implicit raycasting can evaluate smooth normals directly from the scalar-field gradient. Gradient-derived vertex normals could also be incorporated into the explicit pipeline by sampling the volume gradient at each interpolated surface vertex.

**Parameters and Limitations.** The recommended slab thickness is S∈[48,96] for a 512^3^ grid. If $N_{z}-1$ is not divisible by *S*, the final slab is processed as a partial slab without padding, with its upper cell index clamped to $N_{z}-2$. The method targets explicit isosurface extraction and is therefore less suitable for sharp-feature preservation than methods such as Dual Contouring or MC33. In addition, the current implementation is limited to volumes that fit within the memory of a single GPU.

**Unreal Engine Integration Architecture.** The CUDA–OpenGL interoperability path and the Unreal Engine visualization path are implemented as two distinct rendering backends that share the same extracted geometry and scalar-volume representation. In the standalone renderer, CUDA writes vertex positions and normals directly into OpenGL vertex buffers registered through cudaGraphicsGLRegisterBuffer. After the buffers are unmapped, OpenGL renders the generated geometry without a CPU-side copy.

For the Unreal Engine backend, the generated geometry is transferred through a host-side staging interface rather than by directly sharing the OpenGL VBO with Unreal Engine’s rendering hardware interface. Vertex positions, triangle indices, and normals are submitted using a ProceduralMeshComponent, while scalar fields are converted into volume-texture-compatible data for ray-marching visualization. Thus, the zero-copy property applies to the standalone CUDA–OpenGL backend, whereas the Unreal Engine implementation uses an explicit staging transfer.

## Experimental results

### GPU-based isosurface extraction

**Plasma Simulation Dataset.** The input data used in this study are three-dimensional scalar fields generated by a fusion-plasma simulation. Each grid sample stores an electromagnetic potential value defined over a uniform Cartesian grid, and the grid resolutions used in the experiments are 256^3^ and 512^3^ samples.

The scalar values describe the spatial variation of the electromagnetic potential within the computational domain. Isosurfaces are extracted at selected threshold values to reveal regions having the same potential magnitude. Of particular interest are the shape of the plasma boundary, internal high-gradient structures, localized unstable patterns, and the spatial continuity of the extracted surfaces. These features are useful for visually examining morphological changes and identifying regions of interest in the simulated plasma field.

Before visualization, the scalar values are normalized to [0,1] using the minimum and maximum values of each dataset. The isovalue τ is therefore specified in the normalized scalar range. Multiple isovalues are used to examine how the extracted surface topology, geometric complexity, and generated vertex count vary with the selected threshold.

The isosurfaces shown in Fig 1 were generated using the capacity-safe extraction method described in the Proposed Framework section. The evaluation below focuses on geometric continuity, extraction time, rendering performance, and GPU memory usage.

**Fig 1:**
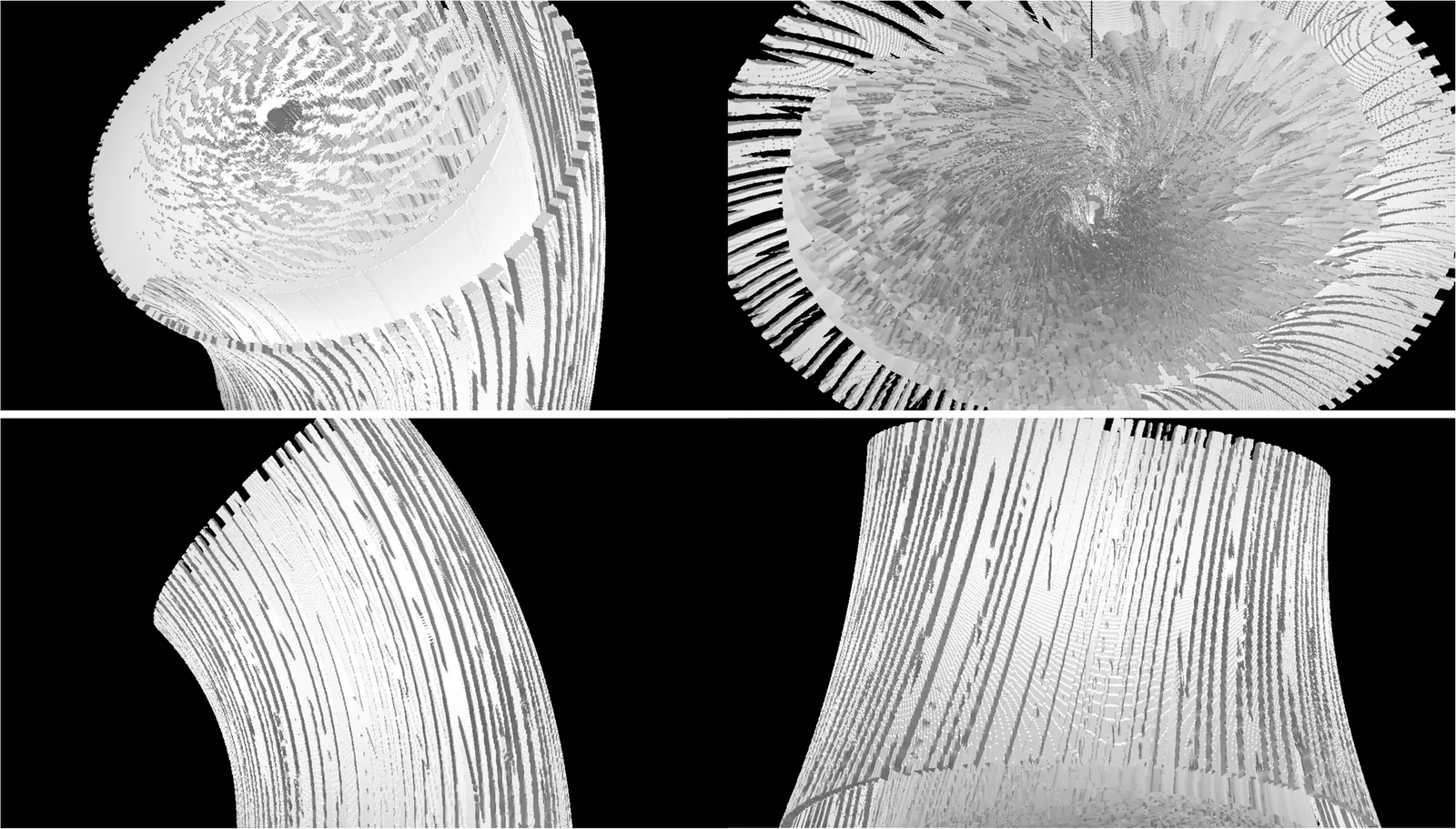
Isosurface extraction of a fusion-plasma subset using the proposed capacity-safe Marching Cubes pipeline. The thin jagged structures mainly reflect scalar-field discretization and flat-shaded polygonal geometry rather than slab-boundary discontinuities.

*Quantitative Performance Evaluation.* To quantitatively evaluate the scalability and interactive performance of the proposed pipeline, we measured the isosurface extraction time, rendering frame rate, and peak GPU memory usage at grid resolutions of 128^3^, 256^3^, and 512^3^. For each resolution, three normalized isovalues, τ=0.35, τ=0.50, and τ=0.65, were evaluated to account for variations in surface complexity and the number of generated vertices.

All experiments were performed on a workstation equipped with an AMD Ryzen Threadripper PRO 3955WX CPU, 256 GB of system memory, and an NVIDIA RTX 3090 GPU with 24 GB of VRAM. Each configuration was executed 100 times after 20 warm-up iterations. The extraction time includes cell classification, prefix-sum computation, exact VBO allocation, and triangle generation. The rendering frame rate was measured after the generated geometry had been transferred to the CUDA–OpenGL interoperable VBO. Peak VRAM usage includes the input scalar volume, intermediate count and offset buffers, and the generated position and normal buffers.

As summarized in Table 2, increasing the grid resolution increases both the number of processed cells and the amount of generated geometry. At 128^3^ and 256^3^, all configurations achieve extraction times below 20 ms and rendering rates above 90 FPS. At 512^3^, the most geometrically complex configuration generates approximately 42.81 million vertices and requires 44.7 ms for isosurface extraction. Nevertheless, the resulting geometry can still be rendered at 28.6 FPS, while the configurations with moderate and high isovalues maintain rendering rates of 36.9 FPS and 49.1 FPS, respectively.

**Table 2:** Performance evaluation of the proposed pipeline across different grid resolutions and isovalues. The extraction time and rendering frame rate are averaged over 100 runs.

Resolution	Isovalue	Vertices (M)	Extraction time (ms)	Rendering rate (FPS)	Peak VRAM (GB)
128^3^	0.35	1.28	5.8	221.4	1.9
128^3^	0.50	0.94	4.9	264.7	1.7
128^3^	0.65	0.61	4.1	312.5	1.6
256^3^	0.35	8.72	19.6	91.8	4.2
256^3^	0.50	6.43	16.8	117.6	3.7
256^3^	0.65	4.21	13.9	148.3	3.2
512^3^	0.35	42.81	44.7	28.6	10.9
512^3^	0.50	31.47	38.2	36.9	9.5
512^3^	0.65	20.86	31.6	49.1	8.0

The results also show that performance depends not only on grid resolution but also on the selected isovalue. Lower isovalues generally produce larger and more complex isosurfaces, increasing the number of generated vertices, extraction time, and memory consumption. In contrast, higher isovalues reduce the geometric output and therefore improve both extraction and rendering performance. Even in the most demanding 512^3^ case, peak VRAM usage remains below 11 GB, demonstrating that the two-pass exact-allocation strategy avoids excessive static buffer reservation and operates within the memory capacity of a single commodity GPU.

These measurements indicate that the proposed pipeline supports interactive visualization across all tested resolutions. Although complete isosurface regeneration at 512^3^ is slower than the subsequent rendering stage, interactive viewpoint navigation and visual analysis remain possible at approximately 29–49 FPS once the VBO has been generated. Moreover, parameter changes such as the isovalue can be processed within tens of milliseconds, allowing responsive exploration of large-scale plasma volumes.

*Visual Analysis.* Fig 1 shows spatially continuous isosurfaces without visible discontinuities at slab boundaries. Fine-scale structures remain identifiable across different viewpoints, while the outer and internal surface regions retain geometric continuity. The jagged appearance of some thin structures primarily originates from the discretization of the input scalar field and the polygonal approximation produced by Marching Cubes. Because the current implementation uses one face normal per triangle, flat shading further emphasizes triangle boundaries and rapid changes in surface orientation. Screen-space aliasing may additionally affect a small number of silhouette pixels, but it is not the primary source of the visible internal patterns. The corresponding extraction time, rendering performance, generated vertex count, and peak GPU memory usage are reported in Table 2.

The method is designed for explicit isosurface extraction and is therefore less suitable for preserving sharp features than approaches such as Dual Contouring or MC33. In addition, the current implementation is limited to volumes that fit within the memory of a single GPU. Future work will investigate out-of-core streaming, tile-based caching, and hybrid extraction methods for improved scalability and feature preservation.

### Surface smoothing and noise reduction

This section evaluates GPU-based smoothing of the electromagnetic-potential scalar field before isosurface extraction (Fig 2). A 256×256×256 uniform Cartesian grid is filtered on the GPU, and the isosurface is subsequently re-extracted from the smoothed field using Marching Cubes.

**Fig 2:**
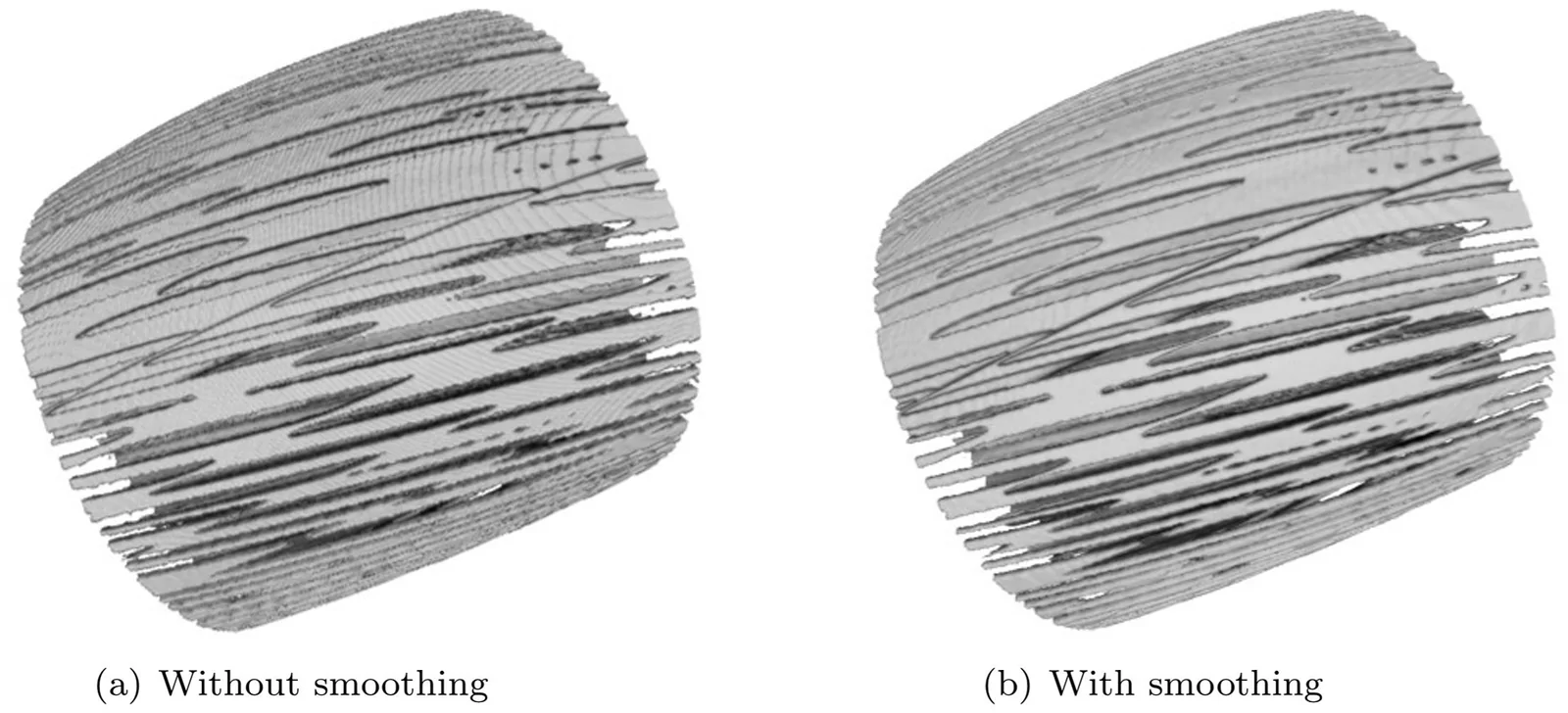
Comparison of isosurfaces extracted before and after GPU-based filtering of the electromagnetic-potential scalar field. The filtering suppresses local high-frequency variations and produces a more spatially coherent surface.

**Input Data and Smoothing Scope.** The input to the smoothing stage is the same electromagnetic-potential scalar field used for isosurface extraction in the preceding experiments. The term “uniform scalar field” refers to the fact that the simulation values are sampled on a regular Cartesian grid with constant spacing along each spatial axis; it does not refer to a separate or artificially generated volume.

The smoothing filter is applied to the scalar-field samples before Marching Cubes isosurface extraction. Thus, the method does not directly average or displace the vertices of an already extracted mesh. Instead, the filtered scalar field is used to recompute the edge-intersection positions during isosurface extraction.

**Implementation Details.** For each scalar sample at (*i*,*j*,*k*), a 3×3×3 box kernel is applied to the valid neighboring samples:


$$\begin{array}{c} \overset{\left(t+1\right)}{\underset{i,j,k}{\tilde{V}}}=(1-\lambda )\overset{\left(t\right)}{\underset{i,j,k}{V}}+\frac{\lambda }{\left|{𝒲}_{i,j,k}\right|}\sum_{(a,b,cin{𝒲}_{i,j,k}}\overset{\left(t\right)}{\underset{a,b,c}{V}}, \end{array}$$ (3)

where ${𝒲}_{i,j,k}$ denotes the valid samples in the local neighborhood and λ=0.35 is the smoothing coefficient. Five iterations are used for the result shown in Fig 2. Boundary samples use only valid neighboring grid locations, without padding.

After filtering, Marching Cubes is performed again on $\tilde{V}$. The position of an isosurface vertex on an intersected grid edge is recomputed as


$$\begin{array}{c} 𝐩={𝐱}_{0}+\frac{\tau -\tilde{V}({𝐱}_{0})}{\tilde{V}({𝐱}_{1})-\tilde{V}({𝐱}_{0})}({𝐱}_{1}-{𝐱}_{0}). \end{array}$$ (4)

Therefore, the smoothing operation affects vertex coordinates indirectly through the filtered scalar values. The triangle connectivity and vertex count may change if the filtered field changes the local Marching Cubes case. Face normals are recomputed from the resulting triangle geometry after extraction, and the choice of normal representation does not affect the scalar-field smoothing operation.

The smoothing example is presented at 256^3^ to allow repeated filtering and side-by-side visual comparison with lower computational and memory cost. This does not indicate that the filter is restricted to 256^3^. The same GPU filtering procedure is applicable to the 512^3^ scalar field, while the 512^3^ experiments in the preceding section are used primarily to evaluate extraction scalability, buffer capacity, and high-resolution performance.

**Visual Result.** As shown in Fig 2, filtering suppresses high-frequency variations in the scalar field and produces a more spatially coherent isosurface. Compared with the unfiltered result, local irregularities in the elongated structures are reduced while the overall plasma morphology is retained. Future work will investigate adaptive and anisotropic kernels for improved feature preservation.

### Detailed plasma visualization in 3D space

Fig 3 presents an isosurface extracted from the 256×256×256 electromagnetic-potential field and rendered from multiple viewpoints. The visualization reveals a geometrically complex core region together with smoother outer structures, allowing fine-scale internal patterns and plasma-boundary morphology to be examined in three dimensions. These results demonstrate that the proposed pipeline preserves the overall spatial organization of the plasma field while supporting interactive visual inspection.

**Fig 3:**
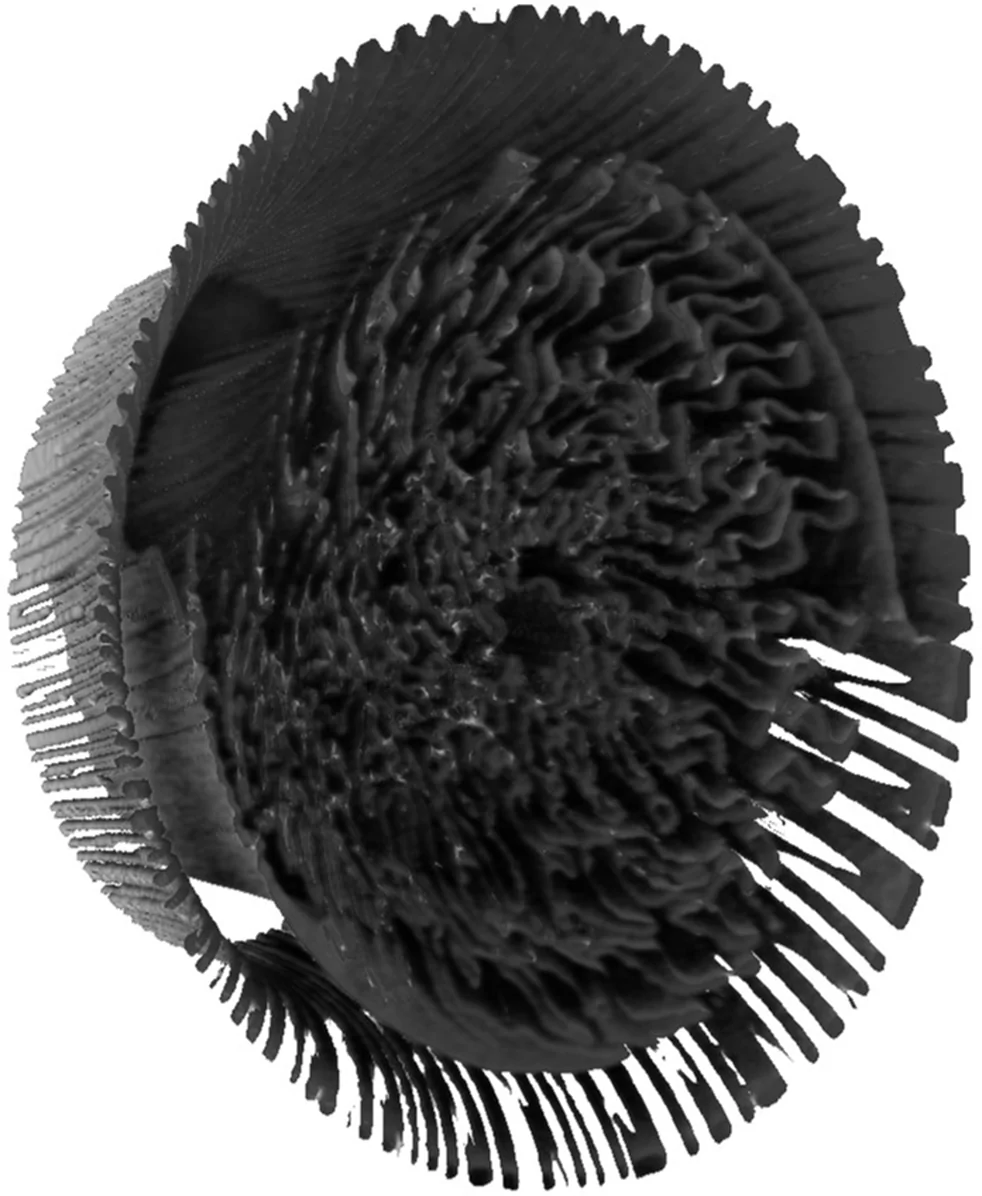
Isosurface extracted from a 256×256×256 electromagnetic-potential field, showing detailed internal and boundary structures in three-dimensional space.

### Failure of the fixed-capacity baseline at 512^3^

Fig 4 shows the result obtained at 512^3^ using the comparison implementation, in which the proposed two-pass exact-allocation strategy is disabled and a fixed-capacity single-pass output buffer is used. The proposed method itself does not fail at this resolution. In the comparison implementation, the generated output exceeds the available buffer capacity, causing overwritten or missing vertex data and producing incomplete geometry.

**Fig 4:**
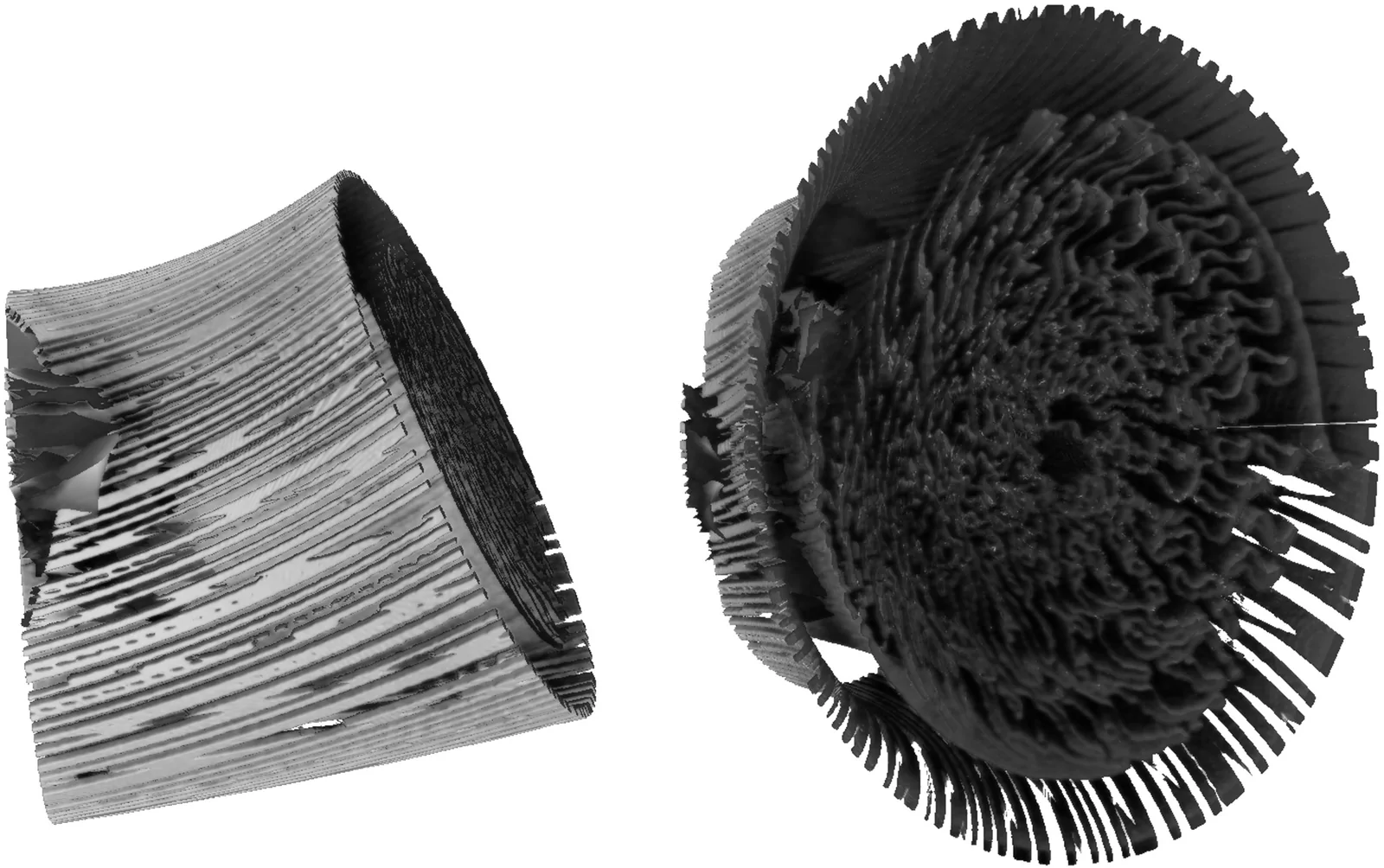
Failure of the fixed-capacity GPU Marching Cubes baseline at a resolution of 512×512×512. Without the proposed two-pass exact-allocation strategy, the output buffer is insufficient, resulting in missing and distorted isosurface geometry. The proposed method remains stable at the same resolution.

The same scalar field and isovalue are used in both configurations. The result therefore isolates the effect of output-buffer management. In contrast, the proposed pipeline determines the exact vertex count before generation and remains stable at the same resolution. Quantitative results for 128^3^, 256^3^, and 512^3^ are reported in Table 2.

### Control of volume visualization integrated with unreal engine

In this study, two types of fusion plasma datasets were used for visualization. The first is grid-based scalar data representing electromagnetic potential values, and the second is trajectory data describing particle motion within the plasma. Among these, the grid-based dataset consists of 3D spatial coordinates (*x*,*y*,*z*) and the electromagnetic potential measured at each position. After several preprocessing steps, this dataset was integrated into an Unreal Engine–based volume rendering pipeline.

For visualization of the grid-based data, we employed the TBRaymarcherPlugin, which supports Ray-Marching–based volume rendering [17]. The plugin loads DICOM and .mhd volume data directly as *Volume Textures* in Unreal Engine and performs real-time rendering using compute-shader-based ray marching. Notably, it includes an efficient implementation of the volume illumination method by Sundén and Ropinski [18], which enhances visual contrast in complex plasma structures.

In addition, the plugin allows users to define transfer functions through Unreal Engine’s Color Curve interface, enabling selective emphasis of specific value ranges or suppression of noise. A *Clipping Plane* can also be applied to inspect cross-sections of the data, which is particularly useful in plasma research where internal structural analysis is essential.

Figs 5 and 6 present visualization results of the grid-based dataset. Fig 5 shows an example in which a Clipping Plane reveals an internal cross-section of the volume, demonstrating that morphological complexity and hierarchical patterns inside the plasma can be intuitively observed. Characteristics such as electromagnetic-potential variation, boundary structures, and spatial pattern formation appear differently depending on the slice, providing useful visual cues for identifying regions of interest.

**Fig 5:**
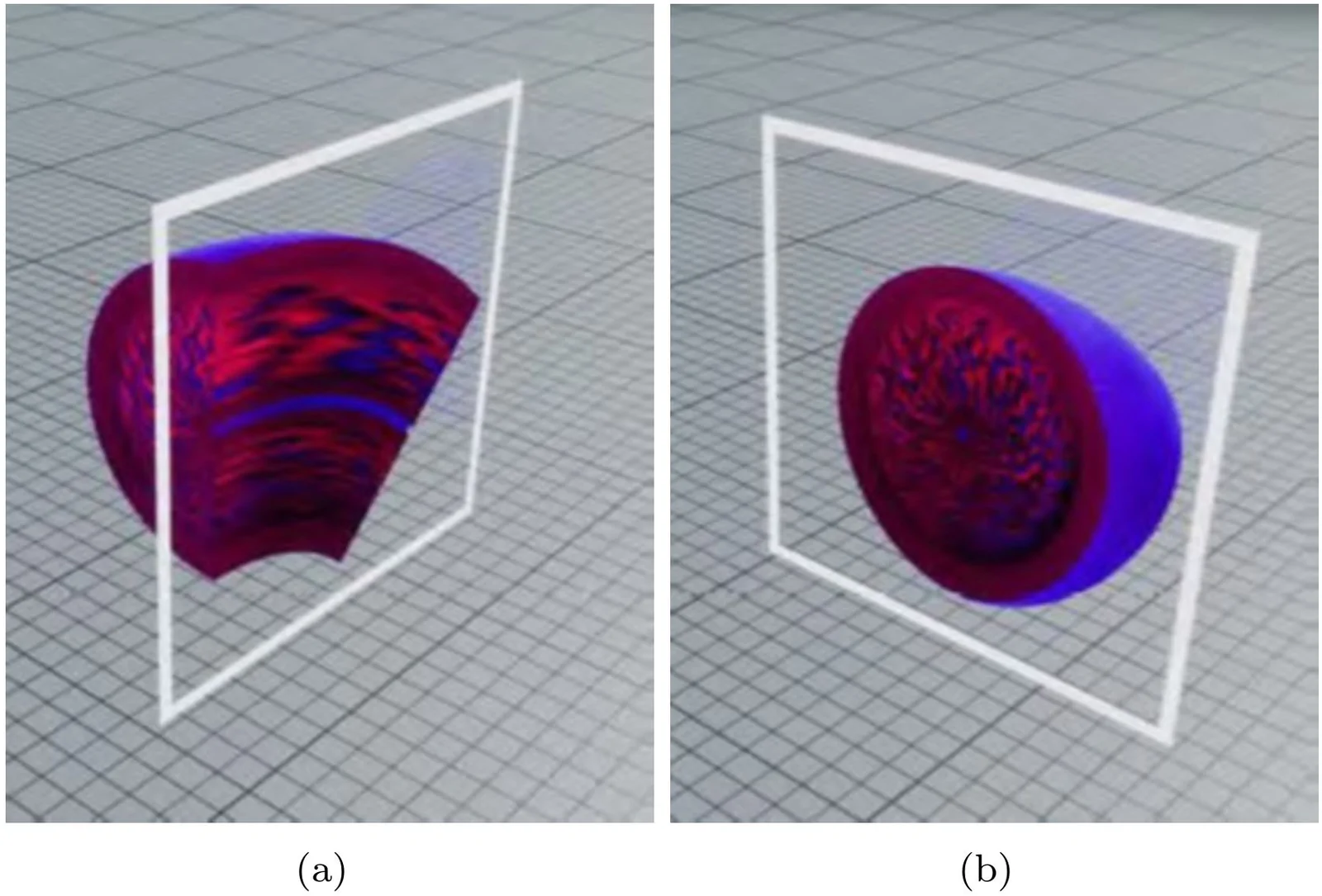
Visualization of the grid-based plasma volume using a clipping plane, enabling inspection of internal structures.

**Fig 6:**
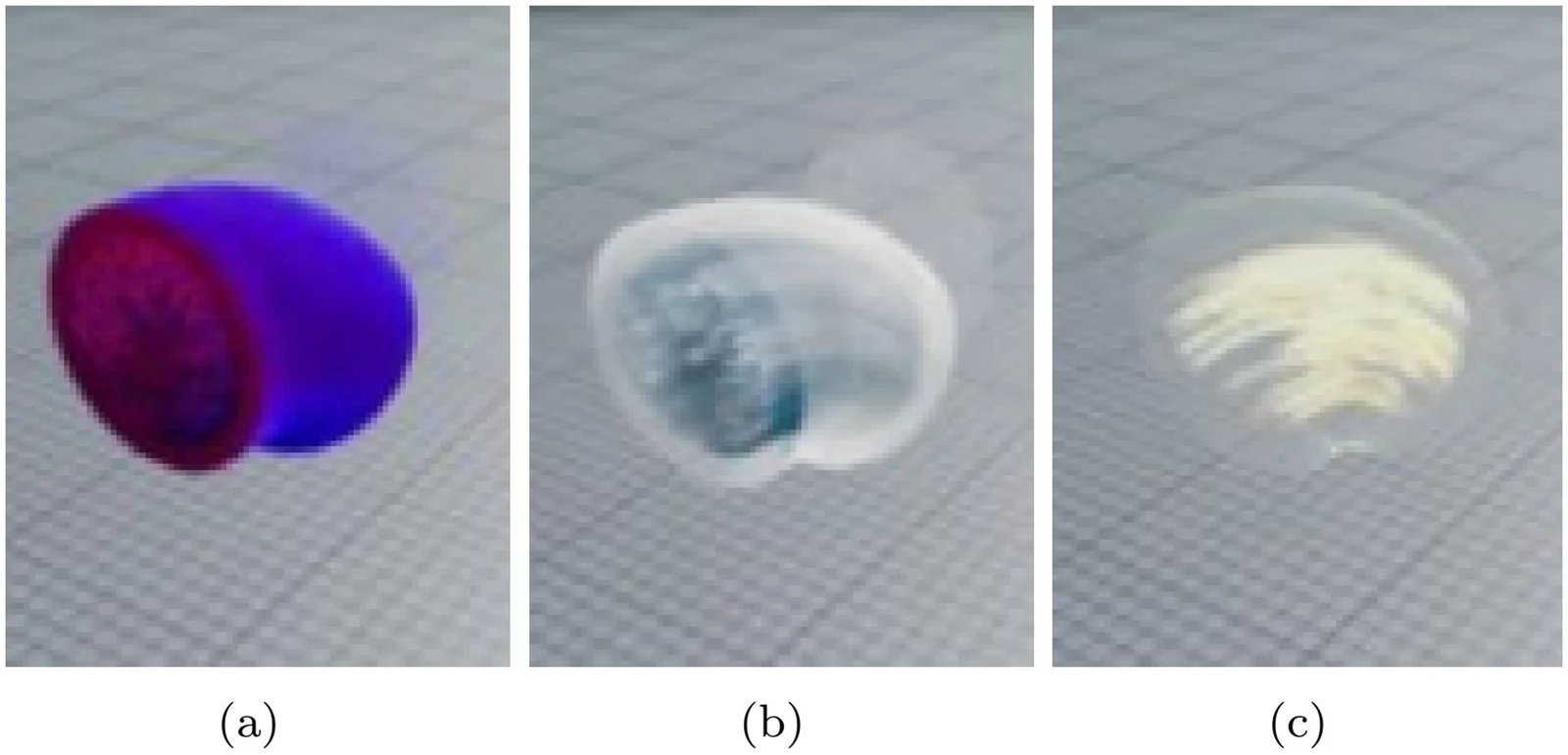
Volume-rendering results obtained using different transfer functions, demonstrating selective emphasis of scalar-value ranges.

Fig 6 compares visualization results obtained using different transfer functions configured through the Color Curve interface (Transfer Functions). Subfigure (a) maps the entire electromagnetic potential range uniformly, while subfigures (b) and (c) selectively highlight specific value ranges–-particularly negative values or regions near a chosen threshold–-thereby making structural features more prominent. By adjusting the Color Curve, users can selectively emphasize specific regions of interest, enabling detailed exploration of complex plasma structures.

Together, transfer-function control and multi-directional clipping allow users to isolate scalar-value ranges and inspect internal structures that would otherwise be occluded in full-volume rendering. Because these parameters can be adjusted interactively in Unreal Engine, regions of interest can be examined from different viewpoints and slicing directions, supporting more focused visual interpretation of the data.

Fig 7 shows an integrated scene in which the proposed isosurface extraction and visualization pipeline is applied to a Tokamak fusion reactor model. A 3D Tokamak structure is placed in the center, and the visualized plasma volume is embedded in the toroidal region. Combined with Unreal Engine’s lighting, materials, and post-processing effects, the plasma appears to emit light from within the actual device. The scene consists of (1) the Tokamak body mesh and (2) environmental lighting and reflection settings provided by Unreal Engine. The plasma is visualized through either ray-marched volume rendering or shaded extracted isosurfaces, with color and transparency adjustments providing clear distinction from surrounding structures. Fig 8 demonstrates interactive manipulation of the extracted plasma structure, including dynamic rotation, slicing, and viewpoint adjustment, enabling global and local structures to be examined from multiple perspectives.

**Fig 7:**
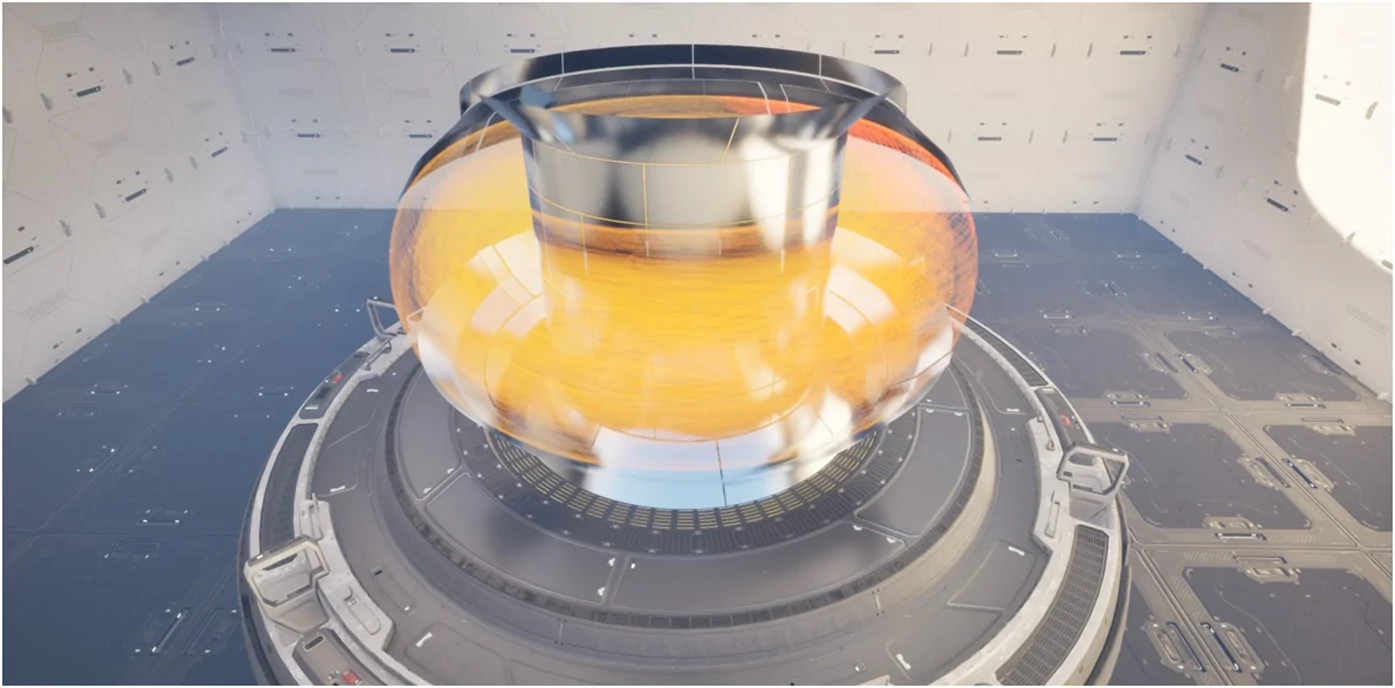
Visualization of a Tokamak fusion reactor scene in Unreal Engine, combining the visualized plasma volume with the reactor model.

**Fig 8:**
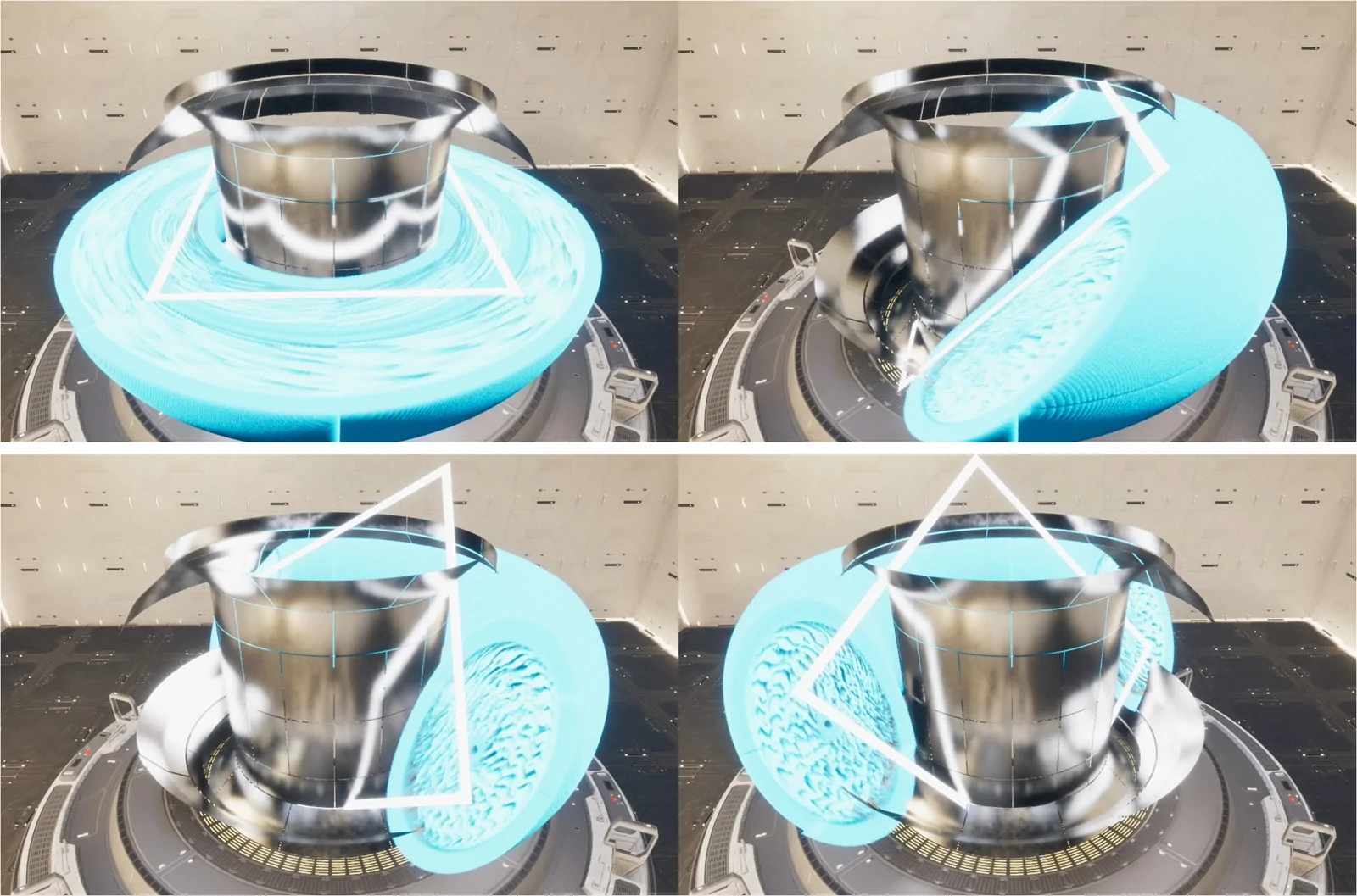
Interactive manipulation of the Tokamak plasma visualization in Unreal Engine, demonstrating dynamic rotation, slicing, and viewpoint adjustment of the extracted isosurface.

These results show that the proposed method extends beyond a standalone volume viewer and can be naturally integrated with complex engineering systems such as fusion reactor digital twins. By visualizing the plasma distribution within the same coordinate system as the Tokamak structure, the system allows the spatial relationship between the plasma boundary, vacuum vessel, divertor, and other internal components to be inspected. This representation can support comparison of plasma shapes under different simulation conditions and examination of potential design changes.

In addition, Unreal Engine supports real-time interactivity (viewpoint control, parameter adjustment, LOD selection, etc.), allowing researchers and engineers to explore different operational scenarios within a single scene. This capability enables repeated examination of the relationship between plasma behavior and reactor geometry. Overall, the example in Fig 8 suggests that the proposed pipeline may serve as a visualization component within fusion-reactor digital twin platforms and operational visualization environments.

### Fusion plasma analysis in a VR environment

Fig 9 shows real-time interaction with the visualized fusion-plasma volume in a virtual reality environment. The system integrates Unreal Engine’s VR interaction framework with the proposed GPU-based isosurface extraction method, allowing users to rotate, translate, scale, and slice the plasma volume directly through hand gestures. Using both hand controllers, the user can grab and manipulate the plasma volume freely in 3D space. These interactions are not merely geometric transforms but are tightly coupled with the underlying volume data, enabling real-time updates of internal structures as the viewpoint or slice changes. When exposing cross-sections, volume clipping is applied simultaneously, enabling intuitive inspection of internal plasma patterns.

**Fig 9:**
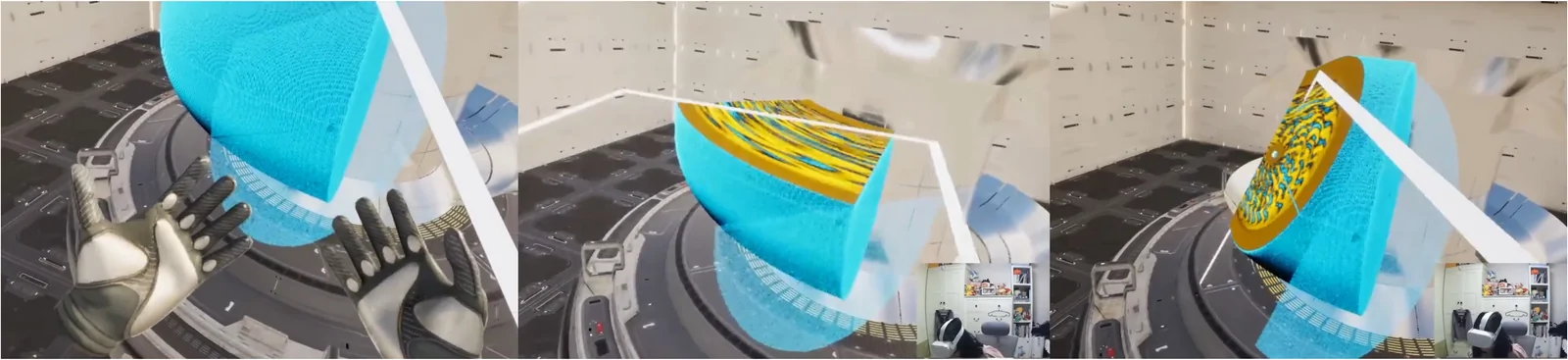
Real-time interaction with the visualized fusion-plasma volume in a virtual reality environment, allowing users to manipulate and inspect internal structures through hand-driven controls.

As shown in Fig 9, VR interaction allows internal electromagnetic-potential structures and spatial variations to be inspected from multiple perspectives. Unlike 2D monitor–based visualization, VR provides stereoscopic depth cues and direct spatial manipulation, which can support the inspection of overlapping surfaces and internal plasma structures. The proposed interactive system supports not only passive observation but also active manipulation and spatial inspection of the visualized plasma data in 3D space. This proof-of-concept suggests potential use in digital-twin visualization, operational support, and education. Owing to the GPU-based isosurface-extraction and rendering pipeline, the VR prototype maintains interactive frame rates during volume rotation, scaling, slicing, and viewpoint manipulation. This result demonstrates the technical feasibility of interactive plasma exploration in a VR environment.

### Particle-based fusion plasma analysis

The particle trajectory data consist of each particle’s unique identifier, the total number of frames, and its 3D position at each frame. In this study, 100 particle trajectories were used to visualize toroidal particle distributions combined with the Tokamak reactor geometry.

**Scalability with Larger Trajectory Sets.** The practical upper limit of trajectory visualization depends on both the number of trajectories and the number of sampled points along each trajectory. For a tube mesh with $N_{t}$ trajectories, $N_{p}$ sampled points per trajectory, and $N_{s}$ radial segments, the number of generated vertices is approximately $N_{t}N_{p}N_{s}$, while the number of triangles is approximately $2N_{t}(N_{p}-1)N_{s}$. For example, 1,000 trajectories with 200 sampled points and eight radial segments generate approximately 1.6 million vertices and 3.18 million triangles.

The current ProceduralMeshComponent-based implementation is suitable for moderate trajectory sets, including configurations containing hundreds of trajectories. Extension to several thousand trajectories is feasible when the geometry is generated once and subsequently rendered as static geometry. However, performance increasingly depends on trajectory length, tube resolution, and update frequency.

For larger datasets, computational and memory costs can be reduced through trajectory decimation, fewer radial segments, distance-based level-of-detail control, and spatial or temporal batching. For continuously updated trajectory sets containing several thousand paths, a GPU-based implementation using compute shaders, structured buffers, or Niagara-based rendering would be more appropriate than repeatedly extracting procedural meshes on the CPU. Thus, the current implementation is intended primarily for interactive analysis of moderate trajectory sets, whereas larger dynamic datasets would benefit from GPU-driven trajectory generation and rendering.

Trajectory visualization was implemented in Unreal Engine using the *Procedural Mesh* technique. Procedural Mesh enables dynamic generation of mesh data at runtime, offering high flexibility for diverse visualization requirements. By defining vertex positions, triangle indices, normals, UV coordinates, and tangents directly in code, only the necessary geometry is generated, minimizing memory usage while maintaining real-time rendering performance.

To visualize each trajectory, the system generates tube-shaped meshes of constant thickness along the particle paths, creating the appearance of continuous lines.

The spiraling and toroidal patterns visible in Fig 10 originate from the centerline trajectories contained in the underlying particle-simulation data. The tube representation does not alter the sampled particle positions or introduce additional curvature; it only assigns a finite radius around each trajectory to improve visibility. Consequently, the tube radius and radial tessellation affect the apparent thickness and smoothness of the rendered paths, while the overall spiral geometry is determined by the simulated trajectories. In densely overlapping regions, the tube representation may visually emphasize the bundled structure, but it does not create the observed trajectory pattern.

**Fig 10:**
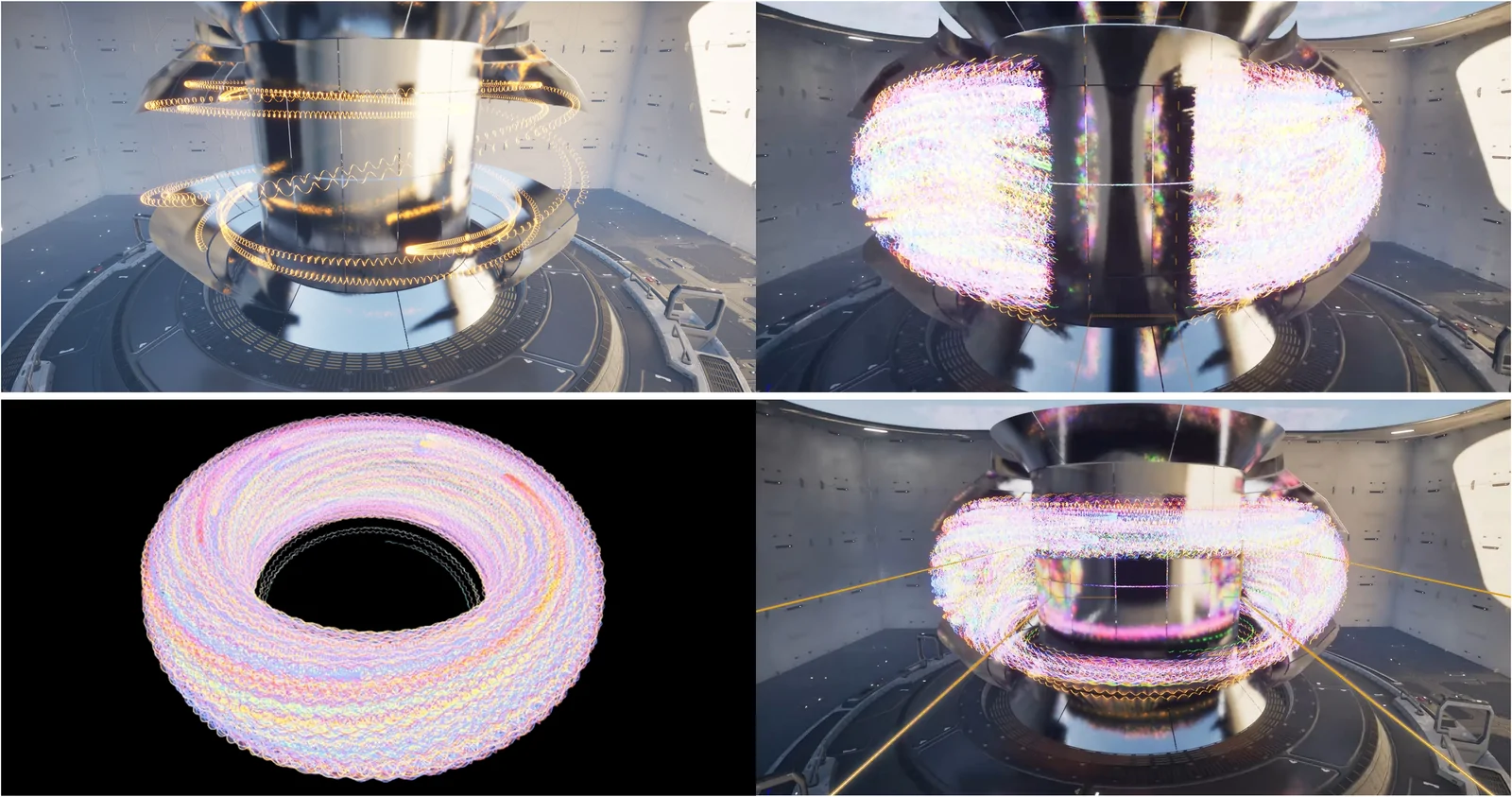
Visualization results of particle trajectory data using Unreal Engine, showing toroidal bundles of trajectories and their spatial relationship to the Tokamak reactor geometry.

This approach conveys the directionality and structure of particle flow more effectively than point-based rendering, enabling intuitive perception of toroidal bundle density and distribution.

Fig 10 shows the particle trajectory visualization results implemented in Unreal Engine. The top image illustrates the overall toroidal structure of particle motion inside the Tokamak. The second image isolates only the trajectories, rendering them as a standalone torus. The third and fourth images apply different clipping planes to highlight how trajectory bundles are spatially arranged relative to the Tokamak geometry and the plasma boundary region. This allows researchers to analyze not only the global distribution of particles but also their spatial relationship to device structures.

The Procedural Mesh–based visualization provides a practical representation for the moderate trajectory sets evaluated in this study while preserving the visual continuity of the flow paths. Combined with clipping planes or controlled camera paths, the system supports focused inspection of selected regions and spatial relationships within the trajectory data.

## Discussion

This work proposes a unified framework comprising *capacity-safe Marching Cubes*, a *CUDA–OpenGL zero-copy pipeline*, and an *Unreal Engine integration* to provide real-time visualization of large-scale fusion plasma data. The framework addresses structural limitations found in traditional CPU-based or offline visualization workflows. Below, we discuss the design implications, computational efficiency, advantages over existing approaches, limitations, and avenues for future research.

**Comparison with Scientific Visualization Toolkits.** ParaView, VTK, and VisIt provide mature scientific-visualization capabilities, including scalable data processing, distributed rendering, advanced filtering, quantitative probing, and support for established scientific data formats. These frameworks are more suitable than the proposed system for large-scale distributed analysis, general-purpose data processing, and production-level scientific workflows.

The purpose of the proposed Unreal Engine integration is therefore not to replace ParaView, VTK, or VisIt. Instead, it provides a complementary visualization environment emphasizing interactive scene composition, real-time rendering, immersive VR exploration, and integration with complex engineering geometry. In particular, Unreal Engine enables plasma volumes, extracted isosurfaces, particle trajectories, reactor geometry, lighting, clipping controls, and interactive user interfaces to be combined within a single spatial environment.

Compared with conventional scientific visualization software, the proposed system offers greater flexibility for constructing immersive digital-twin scenarios and spatially examining the relationship between plasma structures and surrounding reactor components. Conversely, it currently provides fewer quantitative analysis functions, such as statistical probing, distributed-memory processing, and general scientific-filter pipelines. Accordingly, the proposed framework should be regarded as a complementary interactive and immersive front end rather than a replacement for established scientific visualization toolkits.

**Scientific Utility and Role of VR.** The scientific value of the proposed system lies primarily in interactive morphological inspection rather than in replacing numerical analysis. Transfer-function adjustment, clipping planes, isovalue control, and trajectory visualization allow researchers to identify regions of interest, inspect internal plasma structures, and examine spatial relationships between plasma behavior and reactor geometry.

VR further supports spatial understanding by providing stereoscopic depth perception and direct manipulation of three-dimensional structures. This can be useful when examining overlapping surfaces, toroidal trajectories, and internal regions that are difficult to interpret from a fixed two-dimensional display. However, the current VR implementation remains a proof-of-concept visualization interface, and its advantages over desktop-based scientific visualization have not yet been validated through a formal comparative user study. Therefore, we limit our claims to technical feasibility and interactive spatial exploration.

**Explicit Surface Extraction versus Implicit Isosurface Raycasting.** Implicit isosurface raycasting provides several advantages when an isosurface is used only for visualization. It avoids mesh storage and preprocessing, supports multiple isovalues within a single ray-marching pass, and enables smooth normals to be evaluated on the fly from scalar-field gradients, for example using central differences. These properties can make raycasting more efficient than explicit Marching Cubes for direct volume inspection.

The proposed method instead targets workflows in which the isosurface must remain available as explicit geometry for subsequent processing, export, integration with engineering meshes, or use by conventional rasterization and interaction systems. The cost of this flexibility is the additional memory required for vertex and normal buffers and the surface-extraction time incurred whenever the isovalue changes. Therefore, the proposed method should be regarded as complementary to implicit raycasting rather than as a general replacement. A hybrid implementation that switches between direct raycasting and explicit extraction according to the analysis task would be a useful direction for future work.

### Design implications and benefits

The proposed *inclusive slab indexing* and *two-pass capacity-safe allocation* eliminate boundary omissions, cracks, and buffer overflows that frequently occur in grids of size 512^3^ or larger. By computing the exact vertex count in Pass 1 and allocating VBOs accordingly in Pass 2, the method avoids GPU memory contention and atomic increment overheads present in conventional Marching Cubes implementations, ensuring stable performance even at high resolutions.

The CUDA–OpenGL interop–based zero-copy pipeline is another key strength of the proposed method. Because vertex and normal data remain entirely on the GPU, H2D/D2H transfers are eliminated, enabling interactive frame rates even for tens of millions of vertices. This design is particularly beneficial in scientific visualization, digital-twin systems, and XR-based interaction environments where low-latency feedback is essential.

### Integration with game engines and scientific utility

Integration with Unreal Engine provides an additional major contribution. Isosurface extraction, volume rendering, and particle-flow visualization are executed cohesively within a single engine environment. Ray-marching volume rendering, clipping planes, transfer functions, and Procedural Mesh–based trajectory rendering enable simultaneous exploration of plasma boundaries, internal instabilities, and transport phenomena within a unified scene.

Interactive experiments in VR further highlight the utility of the system. Users can directly grab, rotate, and slice the plasma volume to examine structural instabilities and boundary transitions. Such capabilities extend naturally to real-time digital-twin analytics, operational support tools, and immersive educational platforms.

### Limitations

Despite these strengths, several limitations remain. First, the Marching Cubes algorithm used in this work follows the classical MC family, and is therefore less suitable for sharp-feature preservation and handling complex topological ambiguities compared to Dual Contouring or MC33-based schemes. Second, the framework operates entirely within a single GPU’s memory, so extremely large (out-of-core) datasets beyond 512^3^ require integration with tile-caching and streaming techniques. Third, particle tracing currently relies on compute-based processing within Unreal Engine, and additional optimization is needed to support very large particle counts (on the order of millions) efficiently.

### Future work

Several directions can be pursued in future work:

- Out-of-core Marching Cubes: Coupling tile caching with the capacity-safe MC pipeline to support very large plasma datasets (e.g., 1024^3^ and beyond).
- Sharp-feature preserving schemes: Designing hybrid approaches that combine Dual Contouring/MC33 methods with the proposed pipeline.
- NTF/AI-assisted extraction: Leveraging Neural Transportation Fields or diffusion-based correction to further improve iso-surface quality.
- GPU-based large-scale particle advection: Offloading particle advection to an external CUDA path (outside Unreal) to support real-time tracing of hundreds of thousands to millions of particles.
- Full digital-twin integration: Developing an operational interface that couples experimental control parameters with visualization in real time.

## Conclusion

This paper presented a GPU-accelerated framework for interactive visualization of fusion-plasma data. The framework combines capacity-safe GPU isosurface extraction, CUDA–OpenGL interoperable rendering, and an Unreal Engine environment supporting volume rendering, particle trajectories, clipping, and VR-based spatial inspection. The quantitative results demonstrate stable extraction at resolutions up to 512^3^, with rendering rates of approximately 29–49 FPS for the tested high-resolution cases.

The proposed system is intended as a complementary immersive visualization front end rather than a replacement for established scientific-visualization toolkits. Future work will focus on out-of-core data processing, gradient-derived smooth normals, GPU-driven large-scale trajectory rendering, and tighter integration with simulation and digital-twin workflows.

## Supporting information

S1 Code Author-generated source code. The source code used in this study is provided in the Supporting Information.
(RAR)

## References

[1] Lorensen WE, Cline HE. Marching Cubes: A High Resolution 3D Surface Construction Algorithm. In: 1987. 163–9.

[2] Nielson GM, Hamann B. The asymptotic decider: resolving the ambiguity in marching cubes. IEEE Visualization. 1991.

[3] Chernyaev EV. Marching Cubes 33: Construction of Topologically Correct Isosurfaces. CERN. 1995.

[4] Ju T, Losasso F, Schaefer S, Warren J. Dual Contouring of Hermite Data. ACM Transactions on Graphics. 2002;21(3):339–46.

[5] Laramee RS, Hauser H, Doleisch H, Vrolijk B, Post FH, Weiskopf D. The State of the Art in Flow Visualization: Dense and Texture‐Based Techniques. Computer Graphics Forum. 2004;23(2):203–21. doi: 10.1111/j.1467-8659.2004.00753.x

[6] Post FH, Vrolijk B, Hauser H, Laramee RS, Doleisch H. The State of the Art in Flow Visualisation: Feature Extraction and Tracking. Computer Graphics Forum. 2003;22(4):775–92. doi: 10.1111/j.1467-8659.2003.00723.x

[7] McLoughlin T, Laramee RS, Peikert R, Post FH, Chen M. Over Two Decades of Integration‐Based, Geometric Flow Visualization. Computer Graphics Forum. 2010;29(6):1807–29. doi: 10.1111/j.1467-8659.2010.01650.x

[8] Moreland K, VTK-m Team. VTK-m: A Toolkit of Scientific Visualization Algorithms. 2024.

[9] Ayachit U. ParaView Catalyst: Enabling In Situ Data Analysis and Visualization. In: ISAV, 2015.

[10] Whitlock B, Favre JM, Meredith JS. Parallel in situ coupling of simulation with a fully featured visualization system. In: Pacific Graphics. 2011.

[11] Rivi M. In-situ visualization: state-of-the-art and some use cases. PRACE. 2011.

[12] Favre JM. In-situ Visualization with VisIt’s libsim. Tutorial Slides. 2011.

[13] Childs H, et al. A terminology for in situ visualization and analysis systems. UC eScholarship. 2020.

[14] Ayachit U. The SENSEI Generic In Situ Interface. In: ISAV Workshop. 2017.

[15] Grieger D. Interactive in situ visualization. EGICE. 2023.

[16] Ibrahim S, Stitt T, Larsen M, Harrison C. Interactive in situ visualization and analysis using Ascent and Jupyter. In: 2019.

[17] Bazar T. TBRaymarcherPlugin. https://github.com/tommybazar/TBRaymarcherPlugin

[18] Sundén E, Ropinski T. Efficient volume illumination with multiple light sources through selective light updates. In: IEEE Pacific Visualization Symposium, 2015. 231–8.

